# Contributing factors for self-reported HIV in male Peruvian inmates: results of the 2016 prison census

**DOI:** 10.3389/fpubh.2023.1241042

**Published:** 2023-09-25

**Authors:** Carlos Culquichicón, Luis E. Zapata-Castro, Percy Soto-Becerra, Alfonso Silva-Santisteban, Kelika A. Konda, Andrés G. Lescano

**Affiliations:** ^1^CI-Emerge, Center of Emerging Diseases and Climate Change Research, Universidad Nacional de Piura, Piura, Peru; ^2^School of Health Sciences, Universidad Nacional de Piura, Piura, Peru; ^3^Emerge, Emerging Diseases and Climate Change Research Unit, School of Public Health and Administration, Universidad Peruana Cayetano Heredia, Lima, Peru; ^4^Sociedad Científica de Estudiantes de Medicina de la Universidad Nacional de Piura, Piura, Peru; ^5^Center for Interdisciplinary Studies in Sexuality, AIDS and Society, Universidad Peruana Cayetano Heredia, Lima, Peru; ^6^Department of Population and Public Health Sciences, University of Southern California, Los Angeles, CA, United States

**Keywords:** HIV, inmates, Peru, treatment, public health

## Abstract

**Introduction:**

Worldwide, prisons are high-risk settings for the transmission of infectious diseases such as HIV. There is a need to understand the health conditions of prisoners to improve and implement timely strategies for HIV diagnosis and control. Hence, we aimed to identify factors associated with self-reported HIV (srHIV) among Peruvian inmates.

**Methods:**

This study is a secondary data analysis of the First Peruvian Prison Census conducted in 2016. We estimated the prevalence of srHIV in prisoners who were male at birth and the association of srHIV with other social conditions, criminal records, and prevalent health conditions. Nested models identified a multivariable parsimonious model for factors associated with srHIV and yielding prevalence ratios adjusted by the included parameters.

**Results:**

The census surveyed 71,087 male inmates of whom 0.4% reported srHIV (*n* = 305), and 82% of whom were receiving antiretroviral treatment (*n* = 220). In our final multivariable model, srHIV was independently associated with age between 36 and 55 years old vs. >55 years old [parsimonious prevalence ratio (pPR) = 1.98, 95% CI, 0.96–4.08], having a stable partner out of prison (pPR = 1.64, 95% CI, 1.24–2.19), being homosexual (pPR = 4.16, 95% CI, 2.50–6.90), self-report of prevalent tuberculosis co-infection (pPR = 2.55, 95% CI, 1.82–3.58), self-report of prevalent sexually transmitted infections (pPR = 34.49, 95% CI, 24.94–47.70), and self-report of prevalent illicit drug use 30 days before the survey (pPR = 1.91, 95% CI, 1.43–2.56).

**Conclusion:**

Self-reported HIV is associated with multiple social, health and prison risks among Peruvian inmates. Deeply understanding these factors would help to design HIV prevention and control strategies in Peruvian prisons.

## 1. Introduction

Worldwide, prisons are settings with high burden of infectious diseases transmission (1). In 2016, an HIV prevalence of 3.8% was estimated from 10.2 million inmates (2), 15.1% with hepatitis C, 4.8% with chronic hepatitis B, and 2.8% with active tuberculosis (3). In Latin American countries, Brazil reported 12.5–17.4% HIV prevalence in prisons, Argentina 7%, and Uruguay 1.4% (4); however most countries have not studied HIV among prison populations. HIV spreads rapidly in vulnerable groups such as men who have sex with men (MSM), intravenous drug users (IDU) and prisoners (5, 6). Moreover, inmates can be exposed to risk conditions for HIV spread such as IDU, unprotected sex, and tattooing (7).

The history of the HIV epidemic in Peru has two important eras before and after the implementation of the highly active antiretroviral treatment (ART) as a nationwide program in 2004 (8). Ten years after its implementation, its outreach let decreased mortality of HIV patients by 40% (9). Currently, the HIV prevalence is 0.3% for general population and in prisoners is 0.5% (10). In 1995–1996, before the implementation of the ART program, initial studies in key and general populations reported an HIV prevalence of 18.2% among MSM, 7% among patients with co-infection of sexually transmitted infections (STIs), and it ranged from 0.2 to 0.6% among general population (11). Among prisoners, in 1999, HIV infection was reported nationwide in 1.1% of Peruvian prisoners (12). In 2003, another study reported an HIV prevalence of 3% at the largest Peruvian prison, Lurigancho (13). Two screenings conducted by the Peruvian National Penitentiary Institute (INPE in Spanish) estimated an HIV prevalence of 0.4% in 2004, and 0.6% in 2006 (14). There is a lack of studies addressing contributing factors for HIV in prisons, the best knowledge available comes from HIV studies in minority groups in Peru where STIs co-infections, illicit drug use, condomless compensated sex, having a greater number of sex partners and unprotected anal sex are the most important factors of increased risk of HIV among transgender women and MSM of general population (15–17).

In this context, there is a need of well-structured, and reliable studies to continue improving HIV public policies in Peru, especially in prisoners. In 2016, the Peruvian National Institute of Statistics and Informatics (INEI, by its acronym in Spanish), along with the INPE, carried out the First National Prison Census aiming to obtain first-hand prisoners data about demographic characteristics, legal records (convicted or indicted), and self-reported prevalent health conditions (18). We aim to evaluate factors associated with self-reported HIV among Peruvian prisoners to give a clear view of prisoners' current status and drive further directions for prevention and control through adequate HIV treatment in prisons.

## 2. Materials and methods

### 2.1. Study design

This study is a secondary data analysis of the First Peruvian Prison Census led by the Peruvian National Institute of Statistics and Informatics and the National Prison Institute (INEI and INPE, by the acronyms in Spanish) from April 18 to 26, 2016 (18). This cross-sectional assessment was carried out in the 66 prisons of Peru (18). Our secondary analysis focused on males at birth and transgender women given that they have the highest HIV risk of all vulnerable populations and they also represent the largest fraction of the prisoner population.

### 2.2. Population

Overall, the census surveyed 76,180 inmates (coverage rate: 98.8% of prisoners registered in INPE) (18). The remaining 906 (1.2%) prisoners were not surveyed because they were attending hearings or in clinics (18).

### 2.3. Pilot test and survey procedures

In March 2016, INPE and INEI conducted a pilot study with 16 prisoners from the Huacho prison and 14 prisoners from the Chorrillos women's prison in the Lima region, Peru (18). The piloted questionnaire assessed the legibility and pertinence of socioeconomic, health, and incarceration questions. The interviewers conducted surveys using two versions of the questionnaire: a long one of 245 items and a short one of 123 items, both were conducted using tablets and took on average 57 and 35 min, respectively, to complete (18).

After the pilot study, any misleading or confusing questions were removed from both questionnaires or the phrasing was reformulated. The final version of the questionnaire included 134 items and takes ~25 min per participant (18). This validated version of the prison census questionnaire was applied nationwide by 1,600 trained interviewers from the INEI and INPE (18). Surveys were conducted in private rooms, specially conditioned for the study in each prison, where the security of interviewers and the privacy of the participants was guaranteed (18). The final census report does not provide any reliability measures for the validated questionnaire given that no scales were included. Quality control checks were conducted by 66 different survey coordinators, one in each prison (18). Coordinators evaluated daily questionnaire completion and unusual answers, and created backup versions of the datasets which were forwarded to INEI for storage. Finally, INEI conducted consistency evaluations of the collected data by detecting any missing, duplicated, or inconsistent responses.

### 2.4. Measures

We analyzed the self-report of HIV as a binary outcome. It was originally asked to prisoners as follows: “Do you have HIV/AIDS?”. Besides, presence of sexual transmitted infections was asked separately from self-reported HIV as follows: “Do you have any Sexually Transmitted Infections?”. However, without interviewer's clarification, it is possible that respondents didn't realize they are exclusive health conditions. Other important covariates, such as STI, and drug use were asked as follows: “Do you have any Sexually Transmitted Infections?”, “Before admission to prison, did you consume drugs?”. Additional detailed description of the questions can be found in the census questionnaire (19). Multinomial variables of demographics, and criminal records included age, sex at birth, birthplace, highest educational level, stable partner out of prison [Re-categorized variable. (Yes): having a partner or being married, (No): divorced, separated or single], prior incarceration, incarceration as a minor, had an incarcerated relative, legal status (convicted or indicted), sexual identity (reported by the census as heterosexual, bisexual, and homosexual into one category for lesbian/gay/transgender), overcrowded prisons (>20% overcrowd index) (20). Dichotomous variables of health conditions, and risk behaviors included self-report of prevalent health conditions as tuberculosis, sexual transmitted infections (STI), viral hepatitis, depression, anxiety, addiction to psychoactive substances and use of drug, alcohol, or tobacco 30 days before imprisonment (Table 1).

**Table 1:** Prevalence of self-reported HIV (srHIV) and treatment conditions among male Peruvian inmates, 2016.

**Characteristic**	**Males at birth**				***P*-value**
	***N*** = **71,087**		**%**		
srHIV diagnosis	305		0.4		
Ongoing treatment^*^	220		82.1		
Diagnosis prior to incarceration^*^	160		59.7		
	**Ongoing treatment**				
	**No**		**Yes**		
	**n**	**%**	**n**	**%**	
Diagnosis prior to incarceration					0.01
No	27	25.0	81	75.0	
Yes	21	13.1	139	86.9	

* *N* males = 305, missing values of ongoing treatment and diagnosis prior to incarceration = 37.

### 2.5. Data analysis

Our analysis was primarily focused on male prisoners at birth. We evaluated the association between self-reported HIV and its covariates using Chi2 tests for categorical variables and Student's *T*-test for numerical variables.

Poisson family regressions were performed using a log link function and mixed effects multilevel models using prisons as a random-effects level. We estimated nested models following a manual forward selection method to identify covariates associated with self-reported HIV until reaching a parsimonious multivariable model (21). Nested models avoid the possibility of finding spurious associations due to the large sample size available (*n* = 71,087), which might occur by simply calculating effect sizes for each possible covariate. The regression model started without any covariates, and in each successive “round” of selection, the covariate that best explained the variance of srHIV was included in the regression model. These covariates were selected using Bayesian Information Criteria (BIC). Ultimately, nested models identified independent, significantly associated factors with self-reported HIV and fitted the smallest, most parsimonious multivariable regression model that best explained the variance of the outcome. Nested models have been applied before in HIV research (22, 23).Crude and adjusted prevalence ratios (PR) were estimated with 95% confidence intervals (95% CI). All hypotheses were calculated using 5% significance. The analysis was performed using Stata 15.1 ED^®^, and the analysis code is openly available in git-hub (24).

### 2.6. Ethical considerations

The primary study conducted by INEI and INPE verbally consented prisoners to participate in the census and to share their de-identified information in a public dataset for research purposes (18). The 2016 prison census data is publicly available at the INEI website (18, 19). The dataset is coded to maintain privacy of prisoners and does not content any identifiable data. This secondary analysis of de-identified data was approved by Universidad Peruana Cayetano Heredia's Institutional Review Board (SIDISI 103778).

## 3. Results

Among the 71,087 male prisoners at birth, 0.4% (*n* = 305) of them self-reported having HIV. Among them, 82% of males at birth (*n* = 220) were receiving treatment. In 60% of participants (*n* = 160) the diagnoses were received prior to incarceration (Table 1). Also, the proportion of prisoners currently receiving HIV treatment in prison was higher among those diagnosed before incarceration (87%) as compared to those diagnosed during the current prison sentence (75%).

Participants stated that the main reasons for being untreated were no money to afford healthcare, under-awareness of disease severity, and delays in receiving health attention (Table 2).

**Table 2:** Reasons for lack of current HIV treatment, reported by male at birth inmates with srHIV, 2016.

**Reasons**	***n* = 47**	**%**
No money to afford treatment	11	23.4
Considering HIV not serious	8	17.0
Delay of receiving health care	8	17.0
Lack of medicines	6	12.8
Distrust of medical staff	3	6.4
Mistreatment by health care providers	2	4.3
No insurance	2	4.3
Other reasons	7	14.9

There were 71,087 prisoners, with 18–35 years old (55%), elementary education (58%), and no stable partner (51%). Among prison-related characteristics, 17% of prisoners have at least one previous incarceration, and 30% of prisoners had relatives previously in prison (Table 3). Based on the overcrowding index, most of the prisons were overcrowded (73%) (Supplementary material 1).

**Table 3:** Socioeconomic, prison conditions, medical records, and risk behaviors of male Peruvian inmates by self-reported HIV (srHIV) status, 2016.

**Characteristics**	**Males at birth (*****N*** = **71,087)**		**srHIV in male**				*P*-value^*^
			**Without srHIV (*****N*** = **70,782)**		**With srHIV (*****N*** = **305)**		
			***n***	**(%)**	***n***	**(%)**	
**Demographics**							
Age (years)							<0.001
18–35	39,638	55.4	39,227	99.6	143	0.4	
36–55	27,111	37.9	26,784	99.4	151	0.6	
>55	4,820	6.7	4,771	99.8	11	0.2	
Birthplace							<0.001
Lima (Country capital)	19,341	27.0	19,061	99.3	154	0.7	
Other	52,228	73.0	51,721	99.7	203	0.3	
Education							0.029
No education	1,465	2.1	1,436	99.2	12	0.8	
Elementary	40,467	58.0	40,097	99.6	208	0.5	
High school	23,652	33.9	23,483	99.7	92	0.3	
College or higher	4,246	6.1	4,209	99.7	15	0.3	
Stable partner outside of prison^**^							<0.001
Yes	36,405	50.9	36,071	99.7	99	0.3	
No	35,163	49.1	34,711	99.4	206	0.6	
Sexual identity							<0.001
Homosexual	277	0.5	255	92.1	22	7.9	
Bisexual	480	0.9	459	95.8	20	4.2	
Heterosexual	53,335	98.6	52,904	99.6	192	0.4	
**Prison-related**							
Previously incarcerated							<0.001
Yes	46,110	82.9	45,763	99.6	167	0.4	
No	9,521	17.1	9,402	99.2	73	0.8	
No of previous incarcerations							<0.001
0	46,110	82.9	45,763	99.6	167	0.4	
1	5,728	10.3	5,658	99.2	45	0.8	
2	2,149	3.9	2,120	99.3	15	0.7	
3+	1,643	3.0	1,623	99.2	13	0.8	
Incarcerated as a minor							<0.001
Yes	65,254	92.0	64,721	99.6	251	0.4	
No	5,660	8.0	5,585	99.2	43	0.8	
Had an incarcerated relative							<0.001
Yes	21,099	29.8	20,875	99.4	132	0.6	
No	49,758	70.2	49,377	99.7	168	0.3	
Legal status							0.523
Convicted	56,075	78.6	55,594	99.6	243	0.4	
Indicted	15,280	21.4	15,131	99.6	59	0.4	
**Self-reported prevalent medical records**							
Tuberculosis							<0.001
Yes	3,210	4.5	3,129	97.7	73	2.3	
No	67,855	95.5	67,375	99.7	229	0.3	
Sexually transmitted infections							<0.001
Yes	558	0.8	452	81.6	102	18.4	
No	70,580	99.2	70,137	99.7	201	0.3	
Depression							<0.001
Yes	6,378	9.0	6,270	99.1	59	0.9	
No	64,699	91.0	64,230	99.6	244	0.4	
Viral hepatitis							<0.001
Yes	634	0.9	609	96.8	20	3.2	
No	70,400	99.1	69,864	99.6	282	0.4	
Anxiety							<0.001
Yes	5,710	8.0	5,620	99.1	54	1.0	
No	65,348	92.0	64,860	99.6	249	0.4	
Addiction to psychoactive substances							<0.001
Yes	2,091	2.9	2,027	97.7	48	2.3	
No	68,956	97.1	68,438	99.6	254	0.4	
**Self-reported risk behaviors**							
Drug use (30 days before prison)							<0.001
Yes	12,029	16.8	11,849	99.0	119	1.0	
No	59,387	83.2	58,933	99.7	186	0.3	
Alcohol use (30 days before prison)							0.458
Yes	34,387	48.2	34,091	99.6	155	0.5	
No	37,029	51.9	36,691	99.6	150	0.4	
Tobacco use (30 days before prison)							<0.001
Yes	17,226	24.1	17,052	99.4	105	0.6	
No	54,190	75.9	53,730	99.6	200	0.4	

* Pearson Chi-square test.

** With stable partner: married or partner. Without stable partner: single, widower, separated or divorced.

Moreover, self-reported HIV was associated with self-report of prevalent sexually transmitted infections (18.4 vs. 0.3%), self-identified as homosexual (7.9 vs. 0.4%), or bisexual (4.2 vs. 0.4%), prevalent tuberculosis infection (2.3 vs. 0.3%), drug use 30 days before prison (1 vs. 0.3%), having a relative in prison (0.6 vs. 0.3%), not having a stable partner (0.6 vs. 0.3%) and being 36–55 years old compared to 18–35 years old (0.6 vs. 0.4%) (Table 3).

The nested model selected seven covariates in the following order: prevalent sexually transmitted infection, sexuality, drug use 30 days before prison, prevalent tuberculosis, stable partner out of prison, having had an incarcerated relative, and age. Among socioeconomic characteristics, likelihood of self-reported HIV was 98% higher among male at birth prisoners between 36 and 55 years old vs. those >55 years old (pPR = 1.98, 95% CI, 0.96–4.08), and 64% higher in those with an unstable partner (pPR = 1.64, 95% CI, 1.24–2.19). Considering prevalent health conditions, likelihood of self-reported HIV was 155% higher among prisoners with tuberculosis (pPR = 2.55, 95% CI, 1.82–3.58), and it was 34 times greater in male-at-birth prisoners with sexually transmitted infections (pPR = 34.49, 95% CI 24.94–47.70). Regarding risk behaviors, likelihood of self-reported HIV was 91% higher in male-at-birth prisoners with drug use 30 days before imprisonment (pPR = 1.91, 95% CI, 1.43–2.56), and it is four times greater in homosexual prisoners (pPR = 4.16, 95% CI, 2.50–6.90) (Table 4).

**Table 4:** Associated factors with self-reported HIV (srHIV) among male Peruvian inmates, 2016.

**Characteristic**	**srHIV in males**					
	**Bivariate regression (1)**			**Multiple regression parsimonious model (2)**		
	**cPR**	**95% CI**	***P*** **-value**	**pPR**	**95% CI**	***P*** **-value**
**Age**						
18–35 years old	1.58	0.86–2.91	0.144	1.10	0.53–2.27	0.804
36–55 years old	2.44	1.32–2.49	0.004	1.98	0.96–2.08	0.065
>55 years old	Ref.			Ref.		
**Birthplace**						
Lima	1.96	1.56–2.45	<0.001			
Other	Ref.					
**Education**						
No education	2.29	1.04–2.04	0.039			
Elementary	1.35	0.78–2.32	0.281			
High school	1.02	0.58–2.81	0.934			
Undergraduate degree or higher	Ref.					
**Sexual identity**						
Homosexual	21.96	14.36–2.60	<0.001	4.16	2.50–2.90	<0.001
Bisexual	11.55	7.35–2.14	<0.001	2.94	1.74–2.95	<0.001
Heterosexual	Ref.			Ref.		
**Stable partner out of prison**						
No	2.16	1.70–2.74	<0.001	1.64	1.24–2.19	0.001
Yes	Ref.			Ref.		
**Prior incarceration**						
Yes	2.12	1.61–2.79	<0.001			
No	Ref.					
**No of previous incarceration**						
3+	2.19	1.25–2.83	0.006			
2	1.93	1.14–2.27	0.014			
1	2.17	1.56–2.01	<0.001			
0	Ref.					
**Incarceration as a minor**						
Yes	1.98	1.43–2.73	<0.001			
No	Ref.					
**Had an incarcerated relative**						
Yes	1.85	1.43–2.73	<0.001			
No	Ref.					
**Legal status**						
Indicted	Ref.					
Convicted	1.12	0.84–2.49	0.432			
**Tuberculosis**						
Yes	6.73	5.18–2.74	<0.001	2.55	1.82–2.58	<0.001
No	Ref			Ref.		
**Sexually transmitted infections**						
Yes	64.43	51.54–2.54	<0.001	34.49	24.94–2.70	<0.001
No	Ref.			Ref.		
**Viral hepatitis**						
Yes	7.91	5.06–2.36	<0.001			
No	Ref.					
**Depression**						
Yes	2.46	1.86–2.27	<0.001			
No	Ref.					
**Anxiety**						
Yes	2.49	1.86–2.34	<0.001			
No	Ref.					
**Addiction to psychoactive substances**						
Yes	6.26	4.61–2.49	<0.001			
No	Ref.					
**Drug use (30 days before prison)**						
Yes	3.16	2.51–2.97	<0.001	1.91	1.43–2.56	<0.001
No	Ref.			Ref.		
**Alcohol use (30 days before prison)**						
Yes	1.11	0.89–2.39	0.354			
No	Ref.					
**Tobacco use (30 days before prison)**						
Yes	1.65	1.30–2.09	<0.001			
No	Ref.					

(1) Poisson's regression model with robust variance. (2) Poisson's regression model with robust variance within multilevel analysis.

cPR, crude prevalence ratio; pPR, parsimonious model's prevalence ratio.

## 4. Discussion

The first Peruvian prison census estimated 0.4% (*n* = 305) of srHIV among prisoners. Globally, HIV prevalence in prison settings are between two to 10 times greater than community estimations (25, 26). In 1999, the HIV epidemic in Peru initially registered 1.1% infected prisoners (12). Afterwards in 2005, HIV prevalence among inmates was 0.4% (27). These estimations over a decade show the historical progress of the HIV epidemic in Peru. Its decline is likely correlated with multiple interventions designed by the Peruvian HIV control program, which included prisons. Besides, to the natural progress of the HIV pandemic to focus on gender and sexual identity vulnerable groups. Since 2003, Antiretroviral Treatment (ART) is distributed free of charge to patients with a confirmed HIV diagnosis in all Peruvian health facilities, including health facilities within prisons. However, some ancillary tests required to diagnose HIV were not always provided free of charge, and at times that has been a barrier that prevented widespread access to ART in Peru (28).

Special attention should be given to the 13.1% of prisoners living with HIV diagnosis prior to incarceration and without ongoing treatment (*n* = 21). This potentially threatens HIV control in prison settings as without proper treatment and viral suppression, ongoing infection could occur within the prison (29). According to the 2016 Peruvian Ministry of Health's HIV guidelines (29), all prisoners should be screened with HIV rapid test at admission, and all suspected cases or inmates with inconsistent rapid test findings should be confirmed using either 4th generation ELISA test or Western Blot (30). This diagnostic algorithm is helpful but certainly does not totally guarantee the engagement with treatment and viral suppression, especially because completion of this algorithm could take up to 4 months to achieve a confirmed diagnosis, delaying access to potentially life-saving care in the health system (30, 31). Additionally, the implementation of HIV screening and treatment programs remains heterogeneous across Peru, especially in underserved settings such as prisons (30). In practice, not all prisoners are screened for HIV at admittance, especially in prisons outside of Peru's capital, Lima, where lack of institutional resources occurs more frequently (31).

The likelihood of self-reported HIV was 34 times higher among male born prisoners with prevalent sexually transmitted infections (STIs). Currently, STIs are a public health problem worldwide that impose increasing socioeconomic costs, not only because of the burden of disease among a high number of infected people but also the potential impacts on sexual and reproductive health (32). In Peru, the National Strategy for Prevention and Control of STIs, HIV and AIDS implemented nationwide programs in community primary care clinics to deliver comprehensive care on sexual health and education on sexuality, this includes the provision of STIs screening upon admission to prisons (33, 34). In community settings, the HIV program specially seeks to deliver care on sexual health to transgender women and MSM, but there are several barriers as stigma and unawareness of disease that decreased the likelihood of adherence to the program (35). In prison settings, it is important to actively screening for STI and deliver sexual care among transgender women given their transactional sexual practices. Besides, the traditional HIV transmission is enhanced by barriers of the justice system, which fails to allocate them in places where they experience disruption of their own sexual and mental health. Transgender women have reported sexual harassment by prisoners and even security guards, and reported their sexual security vulnerability (36).

The likelihood of self-reported HIV among homosexual and bisexual prisoners were 4- and 2-fold, respectively. Since the beginning of the HIV epidemic in Peru between 12.4 and 22.3% of men-who-have-sex-with-men (MSM) and 30.0% of transgender women are living with HIV, especially in the Lima capital (37, 38). Currently the Peruvian government has not implemented any specific policy to legally recognize transgender women as citizens, however, they are considered a protected group by the Peruvian Ministry of Justice.^1^ This scenario encourages to reframe the current health strategies to deliver care to LGBT groups in Peruvian prisons. Future interventions should target stigmatized individuals in prison and design delivery models in which patients engage to the HIV program through their peers (36, 39). Stigma, discrimination and violence diminish the delivery of sexual care among LGTB+ but especially among transgender women in prison (36, 40). This can discourage people from seeking for sexual health care, and sexuality advice, leading to unsuccess on prevention and control of HIV in prisons (40). A nationwide health policy has been designed to support transgender women with feminization therapy, HIV and STI screening, delivery of condoms and lubricants, and hepatitis B immunization, however, efforts are still needed to equitable implement this policy across Peru but specially in prisons (36, 41, 42). Incarcerated transgender women are placed in male prisons where they experience violence, sexual abuse and frequently have unprotected sexual work to adapt in such disruptive scenario (36). Transgender women prisoners should be relocated to female prisons, which will match with their gender identity and improve their health conditions as well as diminishing the risk of HIV transmission among prisoners. The PMoH and the INPE should implement interventions to address intersectional barriers to health, i.e., gender identity and being incarcerated, and deliver comprehensive care to transgender women in prisons (33, 43).

The likelihood of self-reported HIV was 155% higher among male born prisoners with prevalent tuberculosis (TB) infection. Prisons are associated with higher levels of TB and HIV infection than community population, and may be at higher risk of transmission when control and prevention strategies are not comprehensively implemented. Factors driving TB spread among HIV infected prisoners include overcrowd settings in prison, lack of health care providers, late TB case detection, poor preventative interventions, and sub-optimal treatment of TB cases (44). The implementation response to prevent TB/HIV co-infection represents a valuable opportunity to screen for HIV during tuberculosis evaluations, which would allow stigmatized gender minority groups seek HIV screening under more private conditions (45). This approach would also provide timely screening, safer sex advice, and condom distribution among prisoners and their contacts within prisons.

The likelihood of self-reported HIV was 91% higher among male born prisoners who reported drug use 30 days before imprisonment. The 2017 Peruvian Prison Census report notified that 18,544 inmates used drugs prior incarceration mainly consuming marijuana (58.2%, *n* = 10,855), cocaine (40.1%, *n* = 7,476), alcohol (16.2%, *n* = 12,316), and injectable drugs (0.28%, *n* = 52) (18). These aggregated estimates were reported by the INPE, but they are not available for analysis within prison centers. In 1999 a study reported alcohol (42.6%, *n* = 2,966), cocaine (13%, *n* = 908), injectable drugs (10.3%, *n* = 720), and marijuana (10.2%, *n* = 707) as the most consumed products among prisoners from the Lurigancho prison, the largest prison located in the capital Lima (12). Despite the comparability challenges, the comparison of these two studies might suggest that there have been an increase of marijuana and cocaine consumption, and a reduction in alcohol consumption, while injectable drugs usage remains low. These illicit drugs and alcohol consumption can enable high risk sexual behaviors, potentially leading to unprotected sex and higher risk of HIV infection (46). In the Lurigancho prison, transgender women reported alcohol consumption and unprotected sex with other prisoners, which could lead to HIV transmission. Potential interventions could incorporate alcohol abuse rehabilitation for prisoners, comprehensive mental health support for transgender women, free provision of condoms and sexual risk behavior reduction education to prisoners (36, 47).

It is important to understand our results under the following statements. First, 906 (1.2%) prisoners were not surveyed because they were attending hearings, hospitalized or in outpatient consultation for cancer, dialysis, tuberculosis, and likely HIV, hence there may have been a selection bias to estimate self-reported HIV prevalence (18). Second, self-reported HIV status may potentially lead to a misclassification bias. However, major studies assessing performance of self-reporting HIV status after screening found that 94.1% of patients with prior positive HIV test truly reported their HIV status, as well 99% of the patients who did not have HIV reported their status as negative (PPV = 94.1%, NPV = 87.2%, Se = 51.2%, Sp = 99%) (46). In this sense, using self-reported HIV for estimations provides a reliable estimate of HIV in Peruvian prisoners. Besides, the prevalence of self-reported HIV found in our study is consistent with the HIV prevalence based on serologic testing of 0.5% among Peruvian inmates estimated by ONUSIDA in 2018 (48), and 0.41% (95% CI, 0.28–0.58) estimated by the Peruvian Ministry of Health in 2005 (12). In addition, we believe that these estimates may be biased due to inconsistent access to HIV testing upon admission to prison. Third, the national census survey did not ask specifically about being transwomen. Based on our experience, that many prisoners who self-identified as homosexual or bisexual are transwomen, conflation of sexual and gender identity remains common, but also transwomen may self-identify as heterosexual. Thus, the item on the national census did not allow us properly identify gender minorities, however, we analyzed sexuality data reported in the census and get valid results. Fourth, the prison census didn't specify a study timeframe when asking about reasons for not receiving HIV treatment, which may have led respondents to report their experience in HIV programs at community clinics. However, as 80% of HIV patients were already convicted, they probably have spent many years of incarceration and most likely reported reasons for not receiving HIV treatment from their within-prison experiences.

In conclusion, self-reported HIV prevalence is higher in Peruvian prisons than in community settings and was higher among self-reported sexual and gender minorities. Risk factors for HIV infection in Peruvian prisoners are prevalent sexually transmitted infections, tuberculosis coinfection, being a sexual and gender minority, and drug use. These risk factors should get special attention for framing interventions to timely diagnose, and comprehensively treat prisoners with HIV infection.

## Data availability statement

The original contributions presented in the study are included in the article/Supplementary material, further inquiries can be directed to the corresponding author.

## Ethics statement

The studies involving humans were approved by Cayetano Heredia University Institutional Review Board. The studies were conducted in accordance with the local legislation and institutional requirements. Written informed consent for participation was not required from the participants or the participants' legal guardians/next of kin in accordance with the national legislation and institutional requirements.

## Author contributions

CC, LZ-C, PS-B, AS-S, KK, and AL: study design, data curation, statistical analysis, supervision, and article writing. All authors contributed to the article and approved the submitted version.

## Footnotes

^1^Peruvian Ministry of Justice. *National Human Rights Plan 2018–2021*.
