# Supplementary material for: Contributing factors for self-reported HIV in male Peruvian inmates: results of the 2016 prison census
Source: Front Public Health. 2023 Sep 25;11:1241042. doi: 10.3389/fpubh.2023.1241042 (PMC10560882; doi:10.3389/fpubh.2023.1241042)
Supplement: Supplementary file 1 [file Table_1.docx]

# Supplementary Data

**Supplementary table 1. Prison population, self-reported HIV (srHIV) prevalence, and overcrowd index by prisons in Peru, 2016.**

| **Area** | **Prison** | **Prison population** | **Permitted capacity** | **Overcrowd index (%)** | **srHIV** | | | | |
| --- | --- | --- | --- | --- | --- | --- | --- | --- | --- |
|  |  |  |  |  | **Males** | |  | **Females** | |
|  |  |  |  |  | **n** | **(%)** |  | **n** | **(%)** |
| Amazon | |  |  |  |  |  |  |  |  |
|  | E.P. Pucallpa | 2,047 | 788 | 159.8 | 6 | 0.31 |  | 0 | 0.00 |
|  | E.P. Pto. Maldonado | 711 | 590 | 20.5 | 3 | 0.42 |  | 0 | 0.00 |
|  | E.P. Chachapoyas | 624 | 488 | 27.9 | 6 | 1.00 |  | 0 | 0.00 |
|  | E.P. Bagua Grande | 230 | 60 | 283.3 | 2 | 0.91 |  | 0 | 0.00 |
|  | E.P. Yurimaguas | 157 | 286 | -45.1 | 1 | 0.68 |  | 0 | 0.00 |
|  | E.P. Iquitos | 1,021 | 800 | 27.6 | 10 | 0.98 |  | 0 | 0.00 |
|  | E.P. Mujeres de Iquitos | 64 | 78 | -17.9 | 0 | 0.00 |  | 0 | 0.00 |
|  | E.P. Juanjui | 678 | 654 | 3.7 | 2 | 0.31 |  | 0 | 0.00 |
|  | E.P. Moyobamba | 584 | 544 | 7.4 | 1 | 0.18 |  | 1 | 3.70 |
|  | E.P. Sananguillo | 537 | 636 | -15.6 | 7 | 1.30 |  | 0 | 0.00 |
|  | E.P. Tarapoto | 457 | 280 | 63.2 | 1 | 0.22 |  | 0 | 0.00 |
| Lima capital | |  |  |  |  |  |  |  |  |
|  | E.P. Callao | 3,184 | 572 | 456.6 | 18 | 0.57 |  | 0 | 0.00 |
|  | E.P. Ancon | 2,272 | 2200 | 3.3 | 12 | 0.53 |  | 0 | 0.00 |
|  | E.P. Modelo Ancon II - S.M.V.C. | 1,454 | 1620 | -10.2 | 10 | 0.92 |  | 3 | 0.83 |
|  | E.P. Barbadillo | 1 | . | . | 0 | 0.00 |  | 0 | 0.00 |
|  | E.P. Anexo de Mujeres Chorrillos | 305 | 288 | 5.9 | 0 | 0.00 |  | 1 | 0.33 |
|  | E.P. Mujeres de Chorrillos | 799 | 450 | 77.6 | 0 | 0.00 |  | 28 | 3.63 |
|  | E.P. Virgen de Fatima | 338 | 548 | -38.3 | 0 | 0.00 |  | 3 | 0.90 |
|  | E.P. Virgen de la Merced | 12 | 42 | -71.4 | 0 | 0.00 |  | 0 | 0.00 |
|  | E.P. Lurigancho | 9,509 | 3204 | 196.8 | 73 | 0.77 |  | 0 | 0.00 |
|  | E.P. Miguel Castro Castro | 4,297 | 1142 | 276.3 | 21 | 0.49 |  | 0 | 0.00 |
|  | E.P. Base Naval Callao | 6 | . | . | 0 | 0.00 |  | 0 | 0.00 |
| Central coast | |  |  |  |  |  |  |  |  |
|  | E.P. Huaraz | 1,010 | 350 | 188.6 | 6 | 0.63 |  | 2 | 3.51 |
|  | E.P. Chimbote | 2,311 | 920 | 151.2 | 2 | 0.09 |  | 0 | 0.00 |
|  | E.P. Chincha | 1,326 | 1152 | 15.1 | 6 | 0.45 |  | 0 | 0.00 |
|  | E.P. Ica | 3,919 | 1464 | 167.7 | 12 | 0.33 |  | 1 | 0.33 |
|  | E.P. Canhete | 1,965 | 768 | 155.9 | 9 | 0.46 |  | 0 | 0.00 |
|  | E.P. Huaral | 3,130 | 823 | 280.3 | 22 | 0.70 |  | 0 | 0.00 |
|  | E.P. Huacho | 1,737 | 644 | 169.7 | 8 | 0.48 |  | 1 | 1.67 |
| Central Andes | |  |  |  |  |  |  |  |  |
|  | E.P. Ayacucho | 2,424 | 644 | 276.4 | 1 | 0.04 |  | 0 | 0.00 |
|  | E.P. Huanta | 101 | 42 | 140.5 | 0 | 0.00 |  | 0 | 0.00 |
|  | E.P. Huancavelica | 197 | 60 | 228.3 | 0 | 0.00 |  | 0 | 0.00 |
|  | E.P. Chanchamayo | 570 | 120 | 375.0 | 4 | 0.73 |  | 0 | 0.00 |
|  | E.P. Huancayo | 1,964 | 680 | 188.8 | 3 | 0.15 |  | 0 | 0.00 |
|  | E.P. Mujeres de Concepcion | 31 | 105 | -70.5 | 0 | 0.00 |  | 0 | 0.00 |
|  | E.P. Jauja | 104 | 85 | 22.4 | 0 | 0.00 |  | 1 | 0.97 |
|  | E.P. Satipo | 163 | 50 | 226.0 | 3 | 1.84 |  | 0 | 0.00 |
|  | E.P. Tarma | 84 | 48 | 75.0 | 2 | 2.38 |  | 0 | 0.00 |
|  | E.P. Oroya | 113 | 64 | 76.6 | 0 | 0.00 |  | 0 | 0.00 |
|  | E.P. Huanuco | 2,547 | 1074 | 137.2 | 2 | 0.08 |  | 0 | 0.00 |
|  | E.P. Cerro Pasco | 195 | 96 | 103.1 | 0 | 0.00 |  | 0 | 0.00 |
| Northern coast | |  |  |  |  |  |  |  |  |
|  | E.P. Cajamarca | 1,342 | 888 | 51.1 | 6 | 0.47 |  | 0 | 0.00 |
|  | E.P. Chota | 130 | 65 | 100.0 | 0 | 0.00 |  | 0 | 0.00 |
|  | E.P. Jaen | 377 | . | . | 2 | 0.55 |  | 0 | 0.00 |
|  | E.P. San Ignacio | 79 | 150 | -47.3 | 0 | 0.00 |  | 0 | 0.00 |
|  | E.P. Pacasmayo | 11 | 72 | -84.7 | 0 | 0.00 |  | 0 | 0.00 |
|  | E.P. Trujillo | 4,442 | 1518 | 192.6 | 13 | 0.29 |  | 0 | 0.00 |
|  | E.P. Mujeres de Trujillo | 281 | 160 | 75.6 | 0 | 0.00 |  | 5 | 1.81 |
|  | E.P. Chiclayo | 3,137 | 1143 | 174.5 | 8 | 0.27 |  | 0 | 0.00 |
|  | E.P. Piura | 3,089 | 1370 | 125.5 | 6 | 0.19 |  | 0 | 0.00 |
|  | E.P. Sullana | 91 | 50 | 82.0 | 0 | 0.00 |  | 1 | 1.11 |
|  | E.P. Tumbes | 860 | 384 | 124.0 | 0 | 0.00 |  | 0 | 0.00 |
| Southern coast | |  |  |  |  |  |  |  |  |
|  | E.P. Arequipa | 1,960 | 667 | 193.9 | 7 | 0.36 |  | 0 | 0.00 |
|  | E.P. Mujeres de Arequipa | 150 | 67 | 123.9 | 0 | 0.00 |  | 1 | 0.67 |
|  | E.P. Camana | 261 | 78 | 234.6 | 0 | 0.00 |  | 0 | 0.00 |
|  | E.P. Tacna | 820 | 222 | 269.4 | 0 | 0.00 |  | 0 | 0.00 |
|  | E.P. Mujeres de Tacna | 109 | 40 | 172.5 | 0 | 0.00 |  | 2 | 1.87 |
|  | E.P. Challapalca | 162 | 214 | -24.3 | 2 | 1.23 |  | 0 | 0.00 |
| Southern Andes | |  |  |  |  |  |  |  |  |
|  | E.P. Abancay | 253 | 90 | 181.1 | 1 | 0.43 |  | 0 | 0.00 |
|  | E.P. Andahuaylas | 351 | 248 | 41.5 | 0 | 0.00 |  | 1 | 2.38 |
|  | E.P. Cusco | 2,275 | 800 | 184.4 | 7 | 0.31 |  | 0 | 0.00 |
|  | E.P. Mujeres del cusco | 136 | 62 | 119.4 | 0 | 0.00 |  | 0 | 0.00 |
|  | E.P. Quillabamba | 342 | 80 | 327.5 | 0 | 0.00 |  | 0 | 0.00 |
|  | E.P. Lampa | 136 | 44 | 209.1 | 0 | 0.00 |  | 1 | 0.74 |
|  | E.P. Puno | 576 | 352 | 63.6 | 0 | 0.00 |  | 0 | 0.00 |
|  | E.P. Juliaca | 1,065 | 420 | 153.6 | 0 | 0.00 |  | 0 | 0.00 |
